# Single mutations in the transmembrane envelope protein abrogate the immunosuppressive property of HIV-1

**DOI:** 10.1186/1742-4690-9-67

**Published:** 2012-08-13

**Authors:** Vladimir A Morozov, Alexey V Morozov, Marwan Semaan, Joachim Denner

**Affiliations:** 1 Robert Koch Institute, Nordufer 20, D-13353, Berlin, Germany; 2 Present address: W.A. Engelhardt Institute of Molecular Biology, Vavilova 32, 119991, Moscow, Russia

**Keywords:** HIV, Pathogenesis, Transmembrane envelope protein, gp41, Immunosuppression, Cytokine release

## Abstract

**Background:**

The mechanism by which HIV-1 induces AIDS is still unknown. Previously, synthetic peptides corresponding to the conserved immunosuppressive (isu) domain in gp41 of HIV-1 had been shown to inhibit proliferation and to modulate cytokine expression of immune cells. The question is, whether the viral gp41 can do the same.

**Results:**

We show for the first time that two trimeric forms of glycosylated gp41 released from transfected human cells modulated expression of cytokines and other genes in human PBMCs in the same manner, but at least seven hundred-fold stronger compared to that induced by the isu peptide. Single amino acid substitutions in the isu domain of gp41 introduced by site-directed mutagenesis abrogated this property. Furthermore, replication-competent HIV-1 with a mutation in the isu domain of gp41 did not modulate the cytokine expression, while wild-type virus did. Interestingly, most of the abrogating mutations were not reported in viral sequences derived from infected individuals, suggesting that mutated non-immunosuppressive viruses were eliminated by immune responses. Finally, immunisation of rats with gp41 mutated in the isu domain resulted in increased antibody responses compared with the non-mutated gp41. These results show that non-mutated gp41 is immunosuppressive in immunisation experiments, i.e. *in vivo*, and this has implications for the vaccine development.

**Conclusions:**

These findings indicate that the isu domain of gp41 modulates cytokine expression *in vitro* and suppresses antibody response *in vivo* and therefore may contribute to the virus induced immunodeficiency.

## Background

**Figure 1:**
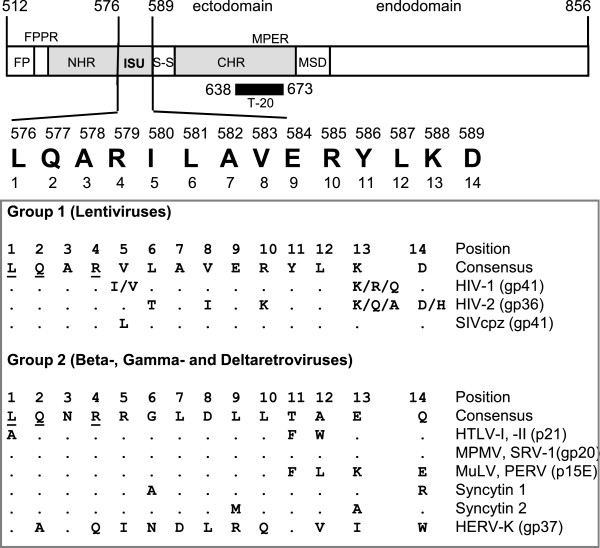
**Functional domains of gp41 of HIV-1, location and alignment of the immunosuppressive (isu) domains of different retroviruses.** FP, fusion peptide; FPPR, fusion peptide proximal region; NHR, N-terminal helical region; ISU, isu domain; S-S, cysteine-cysteine loop; CHR, C-terminal helical region; MPER, membrane proximal external region; MSD, membrane spanning domain. The location of the fusion inhibitor T20 (Enfuvirtide, Fuzeon) is shown as a black bar. Numbering on the top corresponds to the position according the accession number AF324493. The alignment of the conserved sequences of the isu domain of different retroviruses is given in the box (HIV-1, HIV-2, human immunodeficiency virus type −1, -2; SIVcpz, simian immunodeficiency virus of chimpanzees; HTLV-1, -2, human T-lymphotropic virus 1, 2; MPMV, Mason-Pfizer monkey virus; SRV-1, simian retrovirus 1; MuLV, murine leukemia virus; PERV, porcine endogenous retrovirus; syncytin 1 = human endogenous retrovirus HERV-W; syncytin 2 = human endogenous retrovirus HERV-FRD; HERV-K, human endogenous retrovirus-K). Residues common to group 1 and 2 are underlined. In brackets proteins (p) and glycoproteins (gp) and the molecular mass in kDa are indicated.

The acquired immunodeficiency syndrome (AIDS) is a complex disease characterised by a severe immunosuppression on the one hand and an activation of immune cells on the other. The mechanism by which HIV-1 induces the immunodeficiency is still unclear, but it was shown that progression to AIDS correlates with virus load [1]. Many viruses suppress the immune system of the infected host using different strategies. For example herpes viruses incorporate the gene for the immunosuppressive IL-10 into their genome and the measles virus uses its envelope protein for immunosuppression [2-7]. Immunosuppression has been described for many retroviruses, among them feline and murine leukaemia viruses (FeLV, MuLV), and it was shown that inactivated non-infectious retrovirus particles and purified transmembrane envelope (TM) proteins inhibit proliferation of immune cells *in vitro* (for review see [8,9]). Mangeney et al. [10,11] demonstrated that the TM proteins of different retroviruses, including MuLV and the human endogenous virus HERV-FRD (syncytin 2, that is expressed in the human placenta), are immunosuppressive *in vivo*. All retroviral TM proteins contain a highly conserved domain, the so-called immunosuppressive (isu) domain, localised in the C-terminal part of the N-helical repeat (Figure 1). Based on the homology of the isu sequences the retroviruses cluster into two groups. One group is composed exclusively of lentiviruses and the other includes beta-, gamma- and deltaretroviruses. Three residues (L1, Q2 and R4) are common to both groups. Peptides corresponding to this domain (isu peptides) have been shown to inhibit mitogen-triggered proliferation of peripheral blood mononuclear cells (PBMCs) [12-16] and to modulate cytokine release, for example, to increase IL-10 and to decrease IL-2 production [8,17]. Based on these data it was proposed that the gp41 of HIV-1 may play an important role in virus-induced immunosuppression [18]. Changes in the expression of cytokines by recombinant gp41 produced in bacteria have been reported [19-21]; however, since the protein had been produced in *E. coli*, contamination with bacterial endotoxin also inducing cytokine expression cannot be ruled out.

Here we show that gp41 of HIV-1 produced in human 293 T cells (which is glycosylated and endotoxin-free) induced considerable release of IL-10 and IL-6 by PBMCs of healthy donors. In addition, an increase in the expression of MMP-1 (metalloproteinase-1) and a reduction in the expression of FCN1 (ficolin 1) were observed. The influence of ten residues in the isu domain of gp41 on cytokine release was examined by site directed mutagenesis. Single mutations abrogated cytokine modulation. Whereas mutations of six amino acids forming a core region in the isu domain completely abrogated the IL-10 release, amino acids outside the core contributed differently in a donor dependent manner. In the case of IL-6 four of these amino acids are crucial. The donor dependence suggests that genetic host factors (receptor polymorphism or differences in signal transduction?) contributed to the biological activity. In addition, gp41 with a single mutation in the core region induced a better immune response in rats immunised with this protein compared to the wt gp41. Finally and most important, IL-10 released from donor PBMCs was completely abrogated when an infectious virus with a single mutation in the core region was tested, while non-mutated virus efficiently modulated cytokine release.

## Results

### Vectors encoding gp41 variants and characterisation of the gp41 released from transfected human cells

Two expression vectors were designed, one coding for the nearly entire wild-type (wt) gp41 with 25 residues deleted at the C-terminus [amino acids 512–831, GenBank:K03455] and another coding for the ectodomain of gp41 lacking the intracytoplasmic tail and the membrane spanning domain, gp41ΔCT [amino acids 512–695, GenBank:K03455]. Both contained the gp120 signal peptide upstream the gp41 sequences and were later modified in the isu domain by site directed mutagenesis.

**Figure 2:**
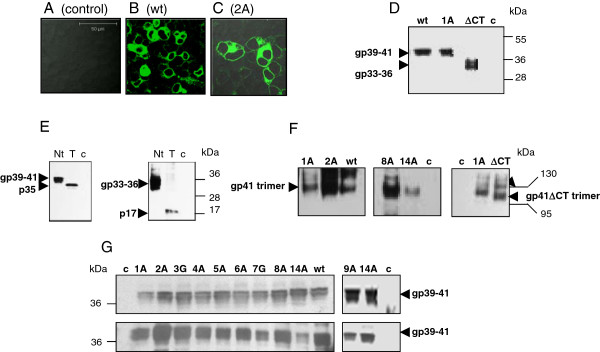
**Expression of recombinant gp41 in human cells and analysis of the released proteins. (A-C)** Immunofluorescence analysis of 293 cells transfected with **(A)** backbone vector pcDNA3.1(−) as negative control, **(B)** expression vector coding for wt gp41, **(C)** and gp41 with the mutation Q2A in the isu domain. Proteins were detected using the mab 2F5. **(D)** Comparison of the wt gp41 and the mutant gp41(L1A) with gp41ΔCT (lacking the cytoplasmatic tail) by SDS-PAGE/Western blot analysis. Proteins in the supernatant were concentrated 20 times using 30 kDa cut-off membranes. **(E)** Evidence for glycosylation of the gp41 and gp41ΔCT produced in 293 cells. Proteins were treated with PNGase F (T), Nt – not treated, c – cells transfected with control vector. p35 is the non-glycosylated form of gp41 and p17 is the non-glycosylated form of gp41ΔCT. **(F)** Evidence for trimerisation of the wt gp41, selected mutants and the gp41ΔCT. After transfection of 293 T cells the proteins were concentrated 20 times as described above and a 4%-20% gradient native PAGE/Western blot analysis was performed. Positions of the trimers (arrow heads) and the molecular markers are given (bars). **(G)** Expression of wt gp41 and gp41 with different mutations in the isu domain in transfected 293 T cells grown in FCS-free medium (upper panel) and proteins recovered from FCS-free medium 48 hours post transfection and concentrated 20 times as described (lower panel). For technical reasons, E9A was analyzed in an additional experiment. A 4-20% gradient SDS-PAGE/Western blot analysis was performed with mab 2F5. Arrow heads indicate the position of gp41. “c” in panels D, E, F and G indicate concentrated supernatant from cells transfected with the backbone vector.

Immunofluorescence analysis of 293 T cells transfected with the vector expressing the wt gp41 revealed the protein on the cell surface and in the cytoplasm (Figure 2). Next 293 T cells grown in FCS-free medium were transfected with vectors coding for the wt gp41 and the gp41ΔCT. The proteins were isolated from the supernatants and concentrated using cut-off membranes as described (Figure 2D).

SDS-PAGE/Western blot analysis of the supernatant from 293 T cells transfected with the vector expressing wt gp41 revealed two glycoproteins of approximately 39 kDa and 41 kDa (Figure 2D). For simplicity the gp39/41 proteins were called wt gp41. Deglycosylation with PNGase F showed that these two glycoproteins have a common protein backbone of 35 kDa, thus, they have some differences in the glycosylation profile (Figure 2E, left panel). The 35 kDa backbone was found also in lysates of transfected cells ( Additional file 1A). Further evidence for glycosylation was obtained by a comparative SDS-PAGE/Western blot analysis of wt gp41 and gp41 with a mutation of the glycosylation site N637T. The mutated N637T protein had a molecular weight of 38 kDa ( Additional file 1B).

Two glycoproteins with a molecular weight of 33 kDa and 36 kDa were found in the supernatant from cells transfected with pgp41ΔCT (Figure 2D). These glycoproteins have a common protein backbone of 17 kDa (p17) as shown by deglycosylation with the PNGase F (Figure 2 E, right panel), indicating a different glycosylation pattern. In transfected cells glycosylation of the p17 occurred in two steps, first p17 undergoes glycosylation to gp25-26 ( Additional file 1C). However, this form was not released and a part of it is likely retained in the endoplasmic reticulum and/or in transport vesicles. The other part undergoes further glycosylation to give rise to the 33–36 kDa glycoproteins eventually found in the cell supernatant ( Additional file 1B, Figure 2E). Since in the cells only traces of gp33-36 were found, they were not retained in cellular compartments, but quickly released. The gp33-36 glycoproteins are called gp41ΔCT.

The conformation of the released proteins was assessed by native gradient PAGE/Western blot analysis. The wt gp41 and gp41ΔCT formed predominantly trimers and also some tetramers or other oligomers (Figure 2F).

### The effect of wild-type gp41 and gp41 with single mutations in the isu domain on cytokine expression

**Figure 3:**
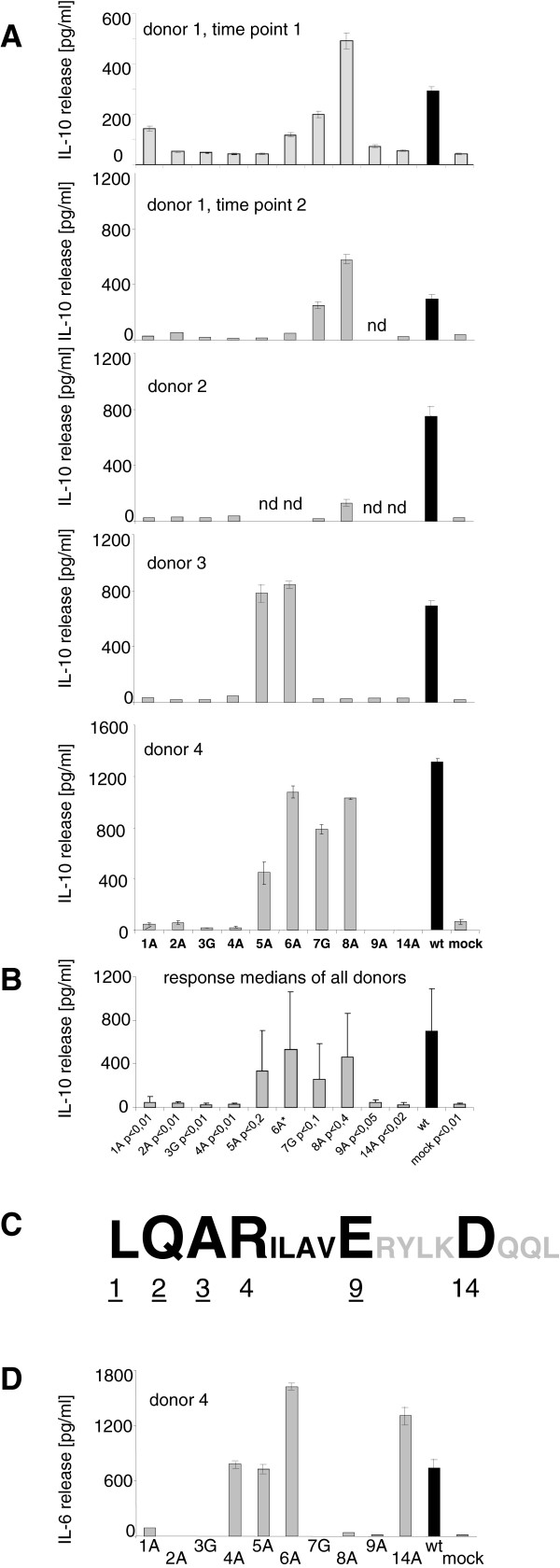
**Cytokine release from PBMCs of healthy donors in response to exposure to the wt gp41 (black) and the mutated gp41 (gray) as estimated by ELISA. (A)** Release of IL-10 from PBMCs of 4 donors measured in triplicates. Wt - wild-type gp41, mock - transfection with the control vector pcDNA3(−), n.d. - not done. PBMCs from donor 1 were tested twice within a three month interval. **(B)** Comparison of medians of IL-10 release from all donor PBMCs and determination of the P values for the influence of each mutant on IL-10 release in comparison with the wt gp41. Error bars represent standard deviations of sample medians, for the P values the Student’s *t* test was used. 6A* - More data are required to make a statistical analysis. **(C)** Schematic presentation of the influence of mutations in the isu domain on IL-10 release, large letters indicate abrogation of IL-10 release (core region), small letters - less inhibition, amino acids given in gray (RYLK) were not investigated. Numbers underlined indicate amino acid residues crucial for both, IL-10 and IL-6 release. **(D)** Release of IL-6 from PBMCs of donor 4 after exposure to wt gp41 and mutated gp41s as estimated by ELISA.

To investigate the effect of wt gp41 on cytokine release, cell supernatant containing 18 ng of the protein was added to donor PBMCs, and after 20 hours the IL-10 release was measured by ELISA. Wt gp41 induced a remarkably high release of IL-10 (Figure 3A, wt). The same result was obtained when the supernatant of cells producing gp41ΔCT was incubated with PBMCs ( Additional file 2), indicating that the biologically active part is located in the ectodomain.

Next we analysed the implication of mutations of ten individual residues in the isu domain of gp41 on cytokine release. Mutations were introduced by site directed mutagenesis into the pgp41(wt), and the mutation 2A (Q577A) was introduced into the pgp41ΔCT. Immunofluorescence analysis of cells producing gp41 with a 2A mutation demonstrated expression of the protein in the cytoplasm and on the cell surface similar to the wt gp41 (Figure 2C). To analyse whether alanine/glycine walking introduces conformational changes in the isu domain, two protein structure prediction programs (DNAStar Lasergene version 10, and PsiPred server, http://http:/bioinf.cs.ucl.ac.uk/psipred/) were used. According to the prediction the substitutions by alanine at positions L1A, Q2A, R4A, I5A, L6A, V8A, E9A and D14A which were called **1A** (L576A), **2A** (Q577A), **4A** (R579A), **5A** (I580A), **6A** (L581A), **8A** (V583A), **9A** (E584A) and **14A** (D589A) do not influence the secondary structure of the isu domain. However, substitutions of alanine by glycine (A3G, A7G) called **3 G** (A578G) and **7 G** (A582G) might induce some disturbance of the α-helical structure. Noteworthy, mutation **14A** (D589A) was introduced to compare the effect of mutations in the isu domain of HIV-1 with that in the isu domain (amino acid at position 14) of a murine gammaretrovirus as well as syncytin 1 (human endogenous retrovirus W that is also expressed in the placenta) [10,11]. Electrophoresis under native conditions demonstrated that four mutated gp41 (mutations 1A, 2A, 8A and 14A) released from transfected cells grown in FCS-free medium formed trimers as was shown for the wt gp41 (Figure 2F).

To prepare proteins for the analysis of cytokine release, 293 T cells were transfected with (i) each of ten constructs encoding gp41 with mutations, (ii) the backbone vector pcDNA3.1(−) (negative control) or (iii) a construct encoding wt gp41 (positive control).

Transfected cells were grown in FCS-free medium to allow protein concentration. An equal expression of the different mutants of gp41 was observed in transfected cells (Figure 2G, upper panel). Supernatants were concentrated twenty times and examined by Western blot analysis. Only minor differences in the amount of released protein (125 ng/ml to 150 ng/ml) were observed (Figure 2G, lower panel). To analyse the influence on cytokine release, the protein amounts were normalised and 18 ng of wt gp41 and 18 ng of each mutated gp41 were added to the PBMCs. PBMCs of each donor were treated with a new batch of proteins; however the stock of endotoxin-free plasmids used for transfection of cells was the same.

Exposure of PBMCs from donor 1 to these ten gp41 mutated in the isu domain demonstrated that the mutations L1A, Q2A, A3G, R4A, E9A and D14A significantly reduced the IL-10 release (Figure 3A). Three months later the PBMCs from donor 1 were tested again using the same panel of gp41 and the response was similar (Figure 3A). Involvement of the six residues L1, Q2, A3, R4, E9 and D14 in IL-10 release was further confirmed using PBMCs from three other donors (2, 3 and 4, Figure 3A). The reduction of the IL-10 release varied from 2 to 60 times and depended on the amino acid residue and the donor of the PBMCs. The difference between the responses to wt gp41 and gp41 with these mutations was statistically significant (Figure 3B, Additional file 3). In contrast, gp41 with the mutations I5A, L6A, A7G and V8A did not always modulate the IL-10 release. Interestingly, gp41 with the mutation V8A even increased the release of IL-10 as found in the case of the PBMCs of donor 1 (Figure 3A). We also analysed double mutants (L1A and R4A, Q2A and L6A). The introduction of a second mutation had no influence on the IL-10 release (not shown). Therefore, six amino acids (L1, Q2, A3, R4, E9 and D14) were critical for IL-10 release (Figure 3C). The mutations L1A, Q2A, A3G, and E9A in gp41 also reduced the IL-6 expression as shown for donor 4 (Figure 3D, Additional file 3B). Interestingly, mutations R4A and D14A reduced the IL-10 release, but had little or no effect on IL-6 release, while mutations A7G and V8A significantly modulated the expression. The reason for this discrepancy is unclear.

Thus, six amino acid residues in the isu domain of gp41 were found to be crucial for the induction of IL-10 release (Figure 3C) and were designated as a core region. Four of these amino acids were also crucial for IL-6 release.

**Figure 4:**
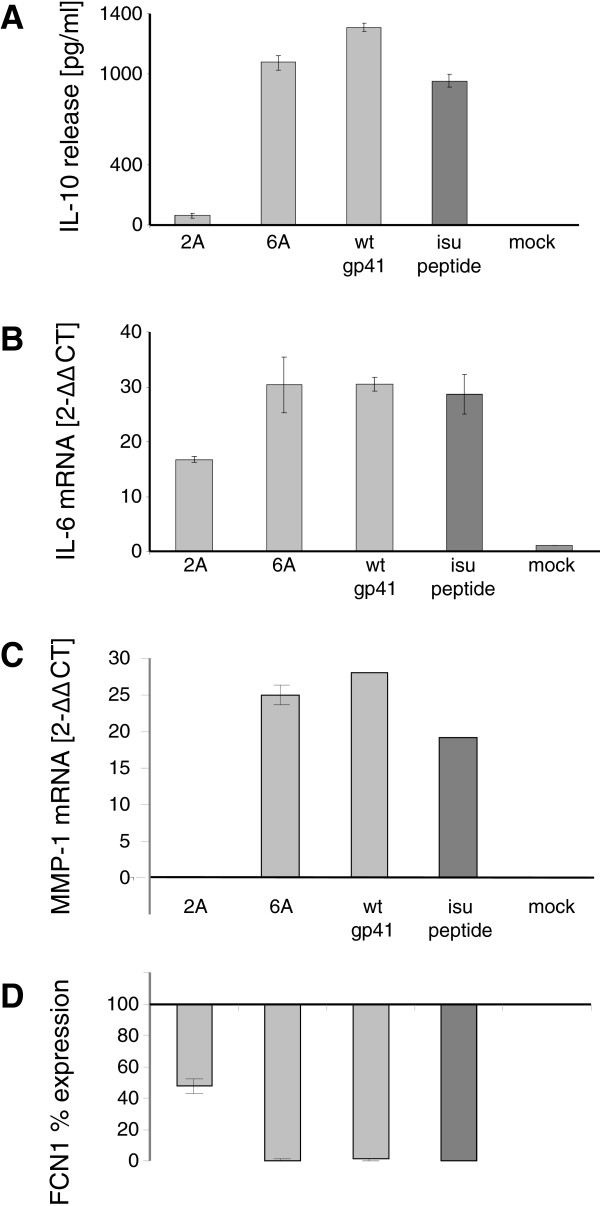
**Comparison of the influence of the wt gp41, gp41 with mutations (2A or 6A) and the isu peptide on cytokine release and gene expression.(A)** IL-10 release from PBMCs of donor 4 after exposure to 18 ng/well of two gp41 mutants (Q2A and L6A) or wt gp41 (light gray), or 12.5 μg/ml isu peptide homopolymer (dark gray). IL-10 was measured in the supernatant by ELISA. Mock supernatants were obtained from 293 cells transfected with the backbone vector. **(B)** Activation of IL-6 and **(C)** MMP-1 transcription as measured by real-time RT-PCR. **(D)** Down regulation of FCN1 expression as measured by real-time PCR and presented as% of the expression induced by the isu peptide. PBMCs were from donor 4.

Direct comparison of cytokine release from PBMCs induced by recombinant gp41 with that induced by homopolymers of a synthetic peptide containing the isu domain showed that approximately 18 ng of recombinant gp41 induced as much IL-10 and IL-6 as 12.5 μg of isu peptide homopolymer as shown by ELISA and real-time PCR (Figures 4A, B; Additional file 4). Thus, a 700–800 times lower concentration of recombinant gp41 is required to modulate the cytokine release compared to the isu peptide. The biologically active conformation of the isu domain in the homopolymer was either rare and/or only partially resembling the native conformation and therefore the homopolymers were significantly less effective.

Using a microarray analysis, the expression of numerous genes besides the IL-6 and IL-10 was found to be up- or down-regulated after treatment with the isu peptide [Denner et al., in preparation]. Among the up-regulated genes was a gene coding for the metallopoteinase-1 (MMP-1). MMP-1 is a member of zinc-dependent proteases that is expressed on the surface of the monocytes and macrophages, and it is essential for the breakdown of extracellular matrix [22]. Among the down-regulated genes was ficolin 1 (FCN1), which is involved in the regulation of the innate immunity. FCN1 binds carbohydrates, activates the lectin pathway of the complement system and mediates clearance of infectious agents by complement dependent phagocytosis [23]. Expression of MMP-1 and FCN1 genes was analysed in PBMCs exposed to recombinant wt gp41, mutated gp41 (2A, 6A) and the polymers of the isu peptide using duplex real-time PCRs (the list of primers and probes is given in Additional file 5).

The wt gp41 suppressed the expression of FCN1 several hundred times more efficiently compared to the isu peptide. The effect of the wt gp41 on the activation of the MMP-1 expression was also much stronger compared with the isu peptide. In contrast, gp41 with a single mutation (Q2A) in the core region of the isu domain was not inducing MMP-1 release or reducing FCN1 expression, and gp41 with a mutation outside the core region (L6A) did not abrogate MMP-1 expression and did not reduce FCN1 expression (Figures 4C, D; Additional file 4).

### Single mutations in gp41 of replication-competent HIV-1 completely abrogated the immunosuppressive effect

**Figure 5:**
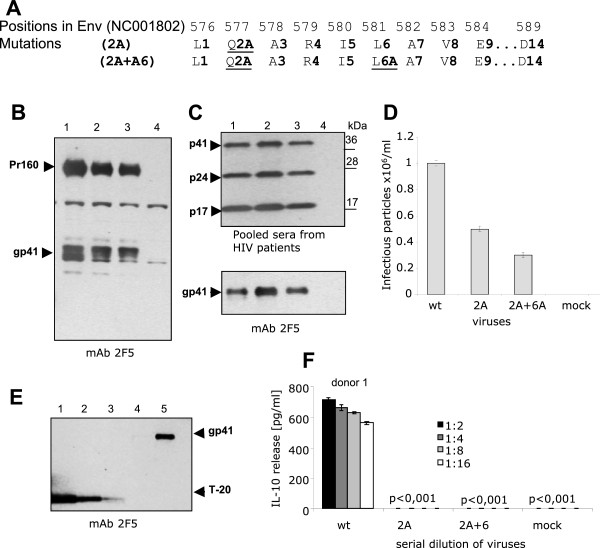
**Replication-competent HIV-1 with mutation in the isu domain does not induce IL-10 release. (A)** Mutations introduced to the isu domain of gp41 HIV-1. The upper numbers started from the first amino acid in Env (accession number AF324493), the lower numbers - with the first amino acid in gp41. The mutated amino acids are underlined. **(B)** Comparison of the virus-specific proteins in transfected cells and **(C)** in the virus centrifuged through a 20% sucrose cushion. 1 - wild-type HIV-1_pNL4-3_; 2 - mutated HIV-1_pNL4-3_ (Q2A); 3 - double mutated HIV-1_pNL4-3_ (Q2A + L6A); 4 - negative control, cells transfected with pcDNA3.1(−). A 4-20% gradient SDS-PAGE/Western blot analysis was performed with the mab 2F5 (B, C lower panel) and with pooled sera from HIV-1 infected individuals (C, upper panel). **(D)** Infectious titers of wt (pNL4-3) and mutated viruses HIV-1pNL4-3 (Q2A) and HIV-1_pNL4-3_ (Q2A + L6A) determined on the reporter TZM-bl cell line. Analysis was performed in triplicates. Mock – supernatant from cells transfected with pcDNA 3.1(−). **(E)** Estimation of the amount of gp41 in virus preparations by SDS-PAGE/Western blot using serial dilutions of T20 as reference. 1–50 ng/lane; 2–25 ng/lane; 3–12.5 ng/lane. Purified HIV-1pNL4-3 was analyzed in two dilutions equivalent to 4 × 10^5^ (Track 4) and 2 × 10^6^ (Track 5) infectious particles per lane. Western blot was performed using the mab 2F5. **(F)** IL-10 release from PBMCs of donor 1 that were exposed to the wt and mutated viruses inactivated by freeze-thawing. Viruses were diluted twofold; the initial concentration of gp41 was 5 ng/well. The p values were determined as described in Methods.

To study the immunosuppressive activity of gp41 in the context of the viral particle, mutations (Q2A and Q2A + L6A) were introduced into gp41 in the infectious molecular clone HIV-1 pNL4-3 (Figure 5A). 293 T cells were transfected with the wt and mutated HIV-1 pNL4-3 and 48 h later the viruses were isolated from the cell supernatants by centrifugation through a 20% sucrose cushion.

No differences in the protein pattern (including gp41) of wild-type and mutated viruses were observed by Western blot analysis (Figure 5B, C). Mutated viruses were infectious as shown by an infectivity assay performed on TZM-bl cells and the titres of the mutated and wt viruses were of the same magnitude (Figure 5D). However, for our experiment it was essential to have the same amount of gp41 in each virus preparation. The concentration of gp41 in the preparations was assessed by Western blot analysis using as reference serial dilutions of the T20 peptide and the mab 2F5 (Figure 5E). To avoid a possible activation of cytokine genes by provirus insertion, the viruses were inactivated by 6 times freeze-thawing. 1.25 ng of viral gp41 with and without mutations in the isu domain were used to induce IL-10 release from 1x10^5^ PBMCs from donor 1. The amount of IL-10 induced by the mutated viruses was approximately 700 fold lower (close to lowest detection limit) compared with that induced by the wt virus (Figure 5F). Comparative analysis of the isu peptide, wt gp41 and gp41 in the virus particles indicated that gp41 in the virus was nearly 1 × 10^4^ times more efficient in induction of IL-10 release compared to the isu peptide and 15 times more efficient than wt gp41 alone. These data indicate that the conformation of the isu domain and possibly the microenvironment of the viral envelope may be important in promoting cytokine modulation. Given the fact that a single amino acid substitution in gp41 inhibits the entire immunosuppressive activity induced by the wt virus, other molecules present in the virus preparation were not involved in cytokine modulation.

### Mutations in the isu domain led to higher antibody responses

Comparison of the immune response against wt gp41 and gp41 with a single mutation (Q2A) in the core region was an approach to analyse gp41-induced immunosuppression *in vivo*. Noteworthy, gp41 is the target of broadly neutralising antibodies such as 2F5 and 4E10 isolated from HIV-1 infected individuals [24] and an important antigen for vaccine development. In this regard, the implication of the isu domain of gp41 on the immune response was also important to examine.

**Figure 6:**
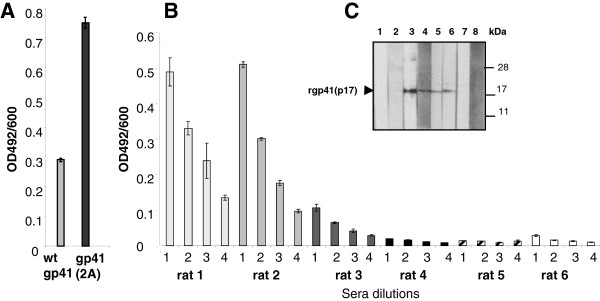
**Enhanced immune response after immunisation with gp41 mutated in the isu domain. (A)** Comparative ELISA of sera from two rats immunised with the wt gp41 and with the mutated gp41(Q2A). T20 (100 ng per well) was used as antigen. **(B)** Comparative ELISA of sera from rats immunised with the mutated gp41ΔCT(Q2A) (rats 1–3) and rats immunised with the wt gp41ΔCt (rats 4–6), all without the cytoplasmatic tail and the membrane spanning domain. 100 ng of T20 per well were used as antigen. Sera were diluted 1–1:200. 2–1:400, 3–1:800, 4–1:1600. **(C)** Western blot analysis of the rat sera using the recombinant ectodomain of gp41 (17 kDa) produced in bacteria. Lane 1, 2 - pre immune sera from rats 1 and 6, lane 3 - mab 2F5 (positive control), lanes 4–6 - sera from rats 1, 2 and 3, all immunized with mutated gp41ΔCt (Q2A), lanes 7–8 - sera from rats 5 and 6, both immunized with wt rgp41ΔCT. The rat sera were diluted 1:200, the mab 2F5 was used at 2 μg/ml, rabbit anti-rat and rabbit anti-human sera conjugated with alkaline phosphatase at 1:2000.

Two rats were immunised subcutaneously with 200 ng of wt gp41 and two rats with mutated gp41(Q2A) followed by three boosts with the same amount of the antigens. An increased antibody response was observed when the mutated gp41 was used for immunisation compared with the wt gp41 (Figure 6A). In a second experiment gp41ΔCT was used for immunisation. This protein demonstrated the same rate of cytokine release when tested *in vitro* to the entire wt gp41 ( Additional file 2). Three rats were immunised with 250 ng of gp41ΔCT and three rats were immunised with 250 ng of mutated gp41ΔCT(2QA) (Figure 6B, C). The immune response to the wt gp41ΔCT was weak, whereas the response to the mutated gp41ΔCT was much stronger as shown by ELISA against the T20 peptide and Western blot analysis using the recombinant ectodomain of gp41 produced in bacteria. These data confirmed and extended the results of the first experiment (Figure 6A).

### Mutations abrogating the immunosuppressive effect were not found in proviruses from HIV-1 infected individuals

Interestingly, the analysis of the isu domain polymorphisms using the Los Alamos HIV and the GenBank databases (more than 2000 HIV-1 sequences were examined) did not reveal mutations of the type L1**A**, Q2**A**, A3**G**, R4**A**, L6**A**, A7**G**, E9**A** and D14**A** in the isu domain of HIV-1 sequences from infected humans (the analysis of 250 sequences is shown in Table 1), indicating that these amino acids are crucial for the viral life cycle.

**Table 1:** Frequency of mutations in the isu domain in proviruses from infected patients

**#**	**aa**	**A**	**D**	**E**	**G**	**F**	**L**	**S**	**Y**	**C**	**W**	**V**	**P**	**H**	**Q**	**R**	**I**	**M**	**T**	**N**	**K**
1	**L**	0	-	-	-	0	*	0	-	-	0	0	1	0	0	2	0	0	-	-	-
2	Q	0	-	0	-	-	0	-	-	-	-	-	9	2	*	94	-	-	-	-	3
3	A	*	0	1	0	-	-	1	-	-	-	1	0	-	-	-	-	-	>100	-	-
4	**R**	0	0	-	7	-	0	0	-	0	0	-	0	0	0	*	0	0	1	-	1
5	I	1	4	-	0	1	5	0	-	-	-	>100	-	-	-	0	*	0	0	0	0
6	L	0	-	-	-	0	*	0	-	-	0	1	3	0	4	2	0	0	-	-	-
7	A	*	1	1	0	-	-	3	-	-	-	1	0	-	-	-	-	-	35	-	-
8	V	3	0	0	2	0	69	-	-	-	-	*	-	-	-	-	92	79	-	-	-
9	**E**	0	1	*	4	-	-	-	-	-	-	0	-	-	0	-	-	-	-	-	5
10	R	4	-	-	22	-	0	9	-	0	0	-	0	0	1	*	0	0	18	-	22
11	Y	0	0	-	-	13	-	0	*	9	-	-	-	3	-	-	-	-	-	0	-
12	**L**	0	-	-	-	0	*	0	-	-	0	0	3	0	0	0	1	0	-	-	-
13	K	27	-	72	97	-	-	15	6	1	41	3	-	41	>100	>100	0	13	37	2	*
14	D	0	*	1	6	-	-	-	3	-	-	1	-	15	-	-	-	-	-	1	-

250 HIV-1 sequences most closely related to the isu domain with the consensus sequence shown in Figure 1 were analyzed for mutations in each position. Numbers indicate the frequency of a substitution at a given position. “-“ indicates that more than one nucleotide substitution is required to change the amino acid, “0” indicates absence of corresponding mutations among the 250 analyzed sequences, * indicates substituted residues. The most conserved residues are given in bold. Please note that no mutations 1LA, 1QA, 4RA, 6LA, 9EA and 14DA as well as no 3AG and 7AG were found.

Further analysis of the sequences from data banks showed that the residues L1, R4, E9 and L12 (not examined) are the most conserved. This observation largely supports our data demonstrating critical residues in the isu domain (Figure 3C). Notable, the residues L1, Q2 and R4 are identical in the isu domain of all retroviruses (Figure 1).

The mutation V8A that enhances the IL-10 release from PBMCs of donor 1 compared with the wild-type gp41 (Figure 3A) was found in viral sequences from infected individuals (Table 1). Using the BLOSUM62 amino acid substitution matrix we found that amino acid exchanges such as Q2R and V8L/I/M described in gp41 from infected individuals were homologous. The evaluation of the disease progression and the cytokine profile in individuals with V8A substitutions in the isu domain might be important for a better understanding of the putative mechanisms of immunosuppression induced by HIV-1 *in vivo*.

## Discussion

Understanding the HIV-1 induced pathogenesis remains a fundamental problem in AIDS research and antiviral therapy. Two principal mechanisms are considered to be involved; first, direct or indirect killing of immune cells and second a general dysregulation of these cells. Based on *in vitro* and *in vivo* experiments we provide evidence that the TM protein gp41 of HIV-1 may be involved in dysregulation of the immune cells, partially by the modulation of the expression of cytokines associated with immunosuppression.

For the first time glycosylated wt and mutated gp41 were produced in human cells. The proteins were recovered from the cell supernatant, characterised and analysed for their immunosuppressive properties. Furthermore, two infectious and replication competent variants of HIV-1 with mutations in the isu domain of gp41 were obtained and examined for their influence on cytokine expression. The systematic and comparative analyses of the properties of the isu domain of gp41 of HIV-1 included (i) glycosylated gp41 released from transfected human cells, (ii) gp41 in the envelope of viral particles and (iii) homopolymers of the isu peptide. All three forms (the gp41 in nanogram and the isu peptide in microgram amounts) induced a significant increase in IL-10 and IL-6 expression. Key residues in the isu domain required for the biological (immunosuppressive) activity were identified. Based on this data we speculate that the putative binding site of the isu domain involved in the induction of IL-10 release is discontinuous (the first 4 amino acids as the first part and position 9 to 14 as the second part). It was shown that immunisation with gp41 mutated in the isu domain resulted in a higher antibody response. Finally, we demonstrated that a single mutation (Q2A) in the isu domain of gp41 of replication-competent HIV-1 completely abrogated the immunosuppressive effect. All together these results indicate that the isu domain is the biologically active domain of gp41 of HIV-1 and the protein may directly interfere with the immune system. These results also shed light on the mechanism of HIV-1 pathogenesis and should be considered when designing gp41-directed vaccines.

An increase in IL-10 and IL-6 release from PBMCs as observed in our experiments was found in the blood of HIV-1 infected individuals [25-29]. Although considered as an immunosuppressive molecule, IL-10 has pleiotropic properties and modulates the function of several adaptive immunity-related cells and has a stimulatory effect on B cells [4]. Noteworthy, a modulation of cytokine release was also shown *in vitro* for the recombinant TM proteins and the isu peptide of gammaretroviruses [17,30] as well as for the human endogenous retrovirus HERV-K (unpublished). Thus, the immunosuppressive effect of the TM proteins seems to be a common property of retroviruses. In this regard it would be of interest to compare the immunosuppressive potential of the TM protein of other retroviruses with that of the gp41 of HIV-1 and the gp36 of HIV-2.

The mechanism of the cytokine modulation induced by gp41 of HIV-1 remains largely unknown. Although several laboratories reported binding of gp41 to the cell surface [31-34], it remains unclear whether there is a receptor(s); and if there is a receptor, how gp41 triggers signal transduction. The following mechanisms may be proposed: (i) The isu domain may interact specifically with a receptor(s) on the cell surface triggering release of cytokines and other factors, which then induce additional changes in gene expression and cytokine release. (ii) The isu domain of gp41 may bind non-specifically to different receptors, e.g., single-spanning transmembrane receptors, for example Toll-like receptors (TLR), inducing dimerization and triggering expression of numerous cytokines, or (iii) it can be a reverse agonist for the receptor(s). In fact, the observed differences in the modulation of cytokine expression in donor 4 (mutations in position L4 and D14 abrogated IL-10 release, but not IL-6 release) may be explained by some differences in the ligand binding site of the receptor(s). In addition a sequence homology between domains in the type I interferons (IFN) and the isu domain of gammaretroviruses and HIV-1 was reported [8,35], and all type I IFNs although different in their sequence bind to a common receptor [36].

Recently a highly conserved region (SWSNKS) in the C-terminal helical region of gp41 was described, which binds to a specific receptor, the globular complement 1q receptor (gC1qR), and induces an increased expression of NKp44L, a cellular ligand for an activating NK receptor on CD4^+^ cells. This may be one reason for the elimination of CD4^+^ cells by NK cells and indicates another mechanism of immunosuppression induced directly by the gp41 protein [37].

The question whether the amount of gp41 in an infected individual is high enough to induce immunosuppression *in vivo* is related to the question where gp41 can be found in the organism. The gp41 with an accessible isu domain may be found on the surface of virus particles after shedding of gp120, in immune complexes, and in debris from dead cells, but the main amount will be found on the surface of infected and virus producing cells in the blood and in lymphoid organs.

Apparently the amount of gp41 in the body of an infected individual is impossible to calculate. Based on our *in vitro* data we attempted to estimate the possible amount of gp41 in the blood of an infected individual. The amount of gp41 in the HIV-1_pNL4-3_ virus preparations was quantified by SDS-PAGE/Western blot analysis using serial dilutions of T20 as a reference (Figure 5E). In our experiment 12.5 ng of gp41 were found in a preparation containing 1x10^6^ infectious virus particles. Thus, one infectious particle was “associated” with 12.5 fg of gp41 (coefficient of association). It is important to underline that in addition to gp41 present in the infectious particles, the preparation contained gp41 from a large number of non-infectious particles. Based on the coefficient of association the following equation was developed:

(1) $$\text{X}=\left(\text{C}\times 10^{\text{n}}\right)\times \text{V}$$

where X is the amount of gp41 in the blood; C is the coefficient of association; 10^n^ is the infectious titre/ml; and V is the volume of the blood in ml. For example, if the infectious titre in an infected individual is 1 × 10^4^/ml, then 5 × 10^7^ infectious virus particles associated with 0.625 μg (0.9 × 10^13^ molecules) of gp41 are present in the blood. Also assuming that 1 × 10^10^ lymphocytes are circulating in the blood, then 9 × 10^2^ molecules of gp41 may interfere with one cell. In our *in vitro* experiments (i) 2 × 10^6^ molecules of wt gp41 produced in human cells, and (ii) 1 × 10^5^ molecules of virus associated gp41 per cell were able to induce changes in the cytokine release. Therefore, the amount of wt gp41 per cell that was used *in vitro* was nearly 2000 times higher and the amount of wt gp41 per cell that was about 100 times higher compared to estimated amount of virus-associated gp41 in the blood of an individual with an infectious titre of 1 × 10^4^/ml. This calculation did not take into account the substantial amount of gp41 on the surface of blood cells, in cell debris and immune complexes. Thus, counting only the virus-associated gp41, an immunosuppressive effect in the blood of the patient comparable to that seen *in vitro* could be achieved if the infectious titre is above 1 × 10^6^/ml.

However, the lymphoid organs have been proposed as the major reservoir of HIV-1 [38]. Numerous molecules of gp41 expressed on the membranes of infected cells in lymphoid organs can actively interact with neighbouring uninfected cells and modulate the cytokine release in a long lasting manner. Thus, taking this point into consideration, the gp41 induced immunosuppression might be significant when the load of infectious virus is much lower than 1 × 10^6^/ml. Since the conformation of gp41 on the cell surface and on virus particles might be different (full size functional gp41-gp120, and thermodynamically stable gp41 stumps, both as trimers or monomers [39]), it still remains to be determined which conformation modulates cytokine release best.

The viruses with mutations in the isu domain were infectious (Figure 5D), and therefore the mutation did not abolish virus replication. In this regard, the fact that certain mutations such as Q2A in the isu domain abrogating the cytokine modulating activity were not found in HIV sequences from infected individuals indicates that non-immunosuppressive viruses might be present only as minor quasispecies and could not represent a majority; otherwise they would be cleared by the immune system. Recently, using a classical laboratory model, a murine retrovirus (MuLV), it was shown that mutations in the isu domain abrogated its ability to suppress the innate and adaptive immune response (NK and CD8^+^ cells) of the immunocompetent host [40]. This mutation also did not influence the replication capacity.

There are still open questions. For example, the virus load in African green monkeys infected with the simian immunodeficiency virus (SIVagm) and in sooty mangabeys infected with SIVsm is as high as in HIV-1-infected individuals [41]. Despite the presence of an isu domain in their TM protein, these SIV do not induce AIDS in their natural host. However, a trans-species transmission of SIVsm to rhesus monkeys resulted in AIDS in the new h-ost, suggesting that the isu domain is functional and that the natural host adapted to it, either by lack of or structural changes in the putative receptor or by disruption of the signal transduction pathway.

Our studies showed that immunisation with gp41 with point mutations in the isu domain induced a better immune response in 4 of 5 rats compared to immunisation with the wt gp41 (Figure 6). It may be possible that conformational changes in the mutated isu domain promote a better immune response, but this was not supported by protein conformation prediction analysis. Similar improved immune responses were reported in mice immunised with the TM protein of the Friend-MuLV mutated in the isu domain in comparison with the wild-type protein; or with the recombinant human syncytin 1 (not immunosuppressive) in comparison with syncytin 2 (immunosuppressive) [10,11].

Viruses developed numerous, often multiple mechanisms allowing suppression of the innate and adaptive immunity [3-7,42]. HIV-1 is not an exception and exploits several strategies to overcome immune responses, for example, down-regulating CD4 and MHC-1 by Nef [43]. In addition, infection of cells by HIV-1 endocytosis may be regarded as a strategy to minimise the contact of the virus with the immune system [44]. Both Tat and gp120 contribute to the deregulation of the immune system, and therefore may be involved in immunosuppression leading to AIDS [45,46]. In this context, it is of interest that the surface envelope protein gp105 of HIV-2 induced a stronger inhibition of T cell proliferation than HIV-1 gp120, in spite of the lower HIV-2 pathogenicity *in vivo,* indicating that gp120 is not the key molecule in inducing immunosuppression [47]. In *in vitro* studies it was shown that gp120 from HIV-1_JR-FL_ and HIV_LAI_ induced IL-10 expression in human monocyte-derived dendritic cells via a mannose C-type lectin receptor, but gp120 from HIV_KNH1144_ did not [48]. Since gp41 is glycosylated, it may interact with such receptors; however the results with the isu peptide homopolymer and the mutations in the isu domain which do not alter glycosylation indicate that glycosylation of gp41 is not involved in cytokine modulation.

It is important to underline that gp41 and gp120 are the first viral proteins that interfere with the immune system during the initial step of infection. Nef and Tat are produced only after infection, and may contribute to the immunosuppression later. In addition, the envelope proteins (especially gp41 anchored in the cell membrane) are expressed on the surface of infected cells and may permanently interact with the immune system.

## Conclusions

In conclusion, gp41 induced a remarkable modulation of cytokine expression in human PBMC. However, recombinant gp41 and HIV-1 particles with single mutations in the isu domain failed to induce cytokine release. Six mutations in the isu domain impaired the cytokine modulation stronger than others. Recombinant gp41 was shown to suppress the antibody response in immunised animals. The gp41-induced cytokine deregulation may contribute to the suppression of the innate and adaptive immunity in HIV-1 infected individuals and may promote virus replication and decrease protection from opportunistic infections. In the course of infection the immunosuppressive effect is likely increasing as a consequence of the increase in virus/gp41 load in the blood and in the lymphoid organs. In addition, interaction of gp120, Tat and Nef with the immune system and an activation of immune cells due to a strong immune response against HIV-1 and other infectious microorganisms take place. All these processes overlap resulting eventually in the clinical picture of AIDS.

## Methods

### Cells

293 T (HEK 293 T) cells were obtained from ATCC (CRL11268), and the reporter TZM-bl cell line was obtained through the NIH AIDS Research & Reference Reagent Program (Cat. # 8129) as contributed by Drs John Kappes and Xiaoyun Wu. The cells were maintained in DMEM supplemented with 10% heat-inactivated fetal calf serum (FCS), antibiotics and L-glutamine.

### Sera and monoclonal antibodies

Pooled human sera from HIV-1 infected individuals (HIV Blot 2.2 kit, MP Diagnostics, Singapore) and 2F5, a human monoclonal antibody against gp41 (recognizing the epitope ELDKWA), kindly provided by Dr. H. Katinger (Polymun, Vienna, Austria), were used.

### Design of expression vectors, site-directed mutagenesis and plasmid purification

The pNL4-3 molecular clone [catalog # 114, GenBank:AF324493] was obtained from the NIH AIDS Research & Reference Reagent Program. The gp41 PCR amplicon was obtained from pNL4-3 by PCR amplification using primers with introduced XhoI and XbaI restriction sites (underlined): forward primer starting with position 7725 (XhoI) 5’- gagtggtgcagagagaaactcgagcagtggg and reverse primer starting with 8722 (XbaI) 5’- agctgcttgttatacttctagaaccctat. The amplicon was treated with XhoI and XbaI endonucleases (Fermentas Life Sciences, Germany) and ligated into the pcDNA3.1(−) (Invitrogen Life Technologies, Carlsbad, CA, USA) together with the gp120 signal peptide using T4 DNA ligase (Fermentas). The signal peptide was introduced as a synthetic sequence with NheI and XhoI restriction sites (5’ctagcatgagagtgaaggagaagtatcagcacttgtggagatgggggtggaaatggggcaccatgctccttgggatattgctcgag3’) and ligated to the pcDNA3.1(−) previously cleaved with NheI and XhoI. The vector (p wt gp41) expressed the gp41 with a 25 amino acid deletion at the cytoplasmatic tail (designated wt gp41). Truncated gp41, designated gp41ΔCT, lacking the cytoplasmic tail and the membrane spanning domain (amino acids 512–695) [GenBank:NC001802], was expressed from the pgp41ΔCT vector designed in the following way: HIV-1pNL4-3 was amplified by PCR with primers containing the XhoI and XbaI restriction sites (underlined): foreword primer starting with position 7725 (XhoI) 5’- gagtggtgcagagagaaactcgagcagtggg and reverse primer starting with 8264 (XbaI) 5’- ttttatctagaacagccaatttg. The amplicon was digested with XhoI and XbaI endonucleases (Fermentas) and ligated into the pcDNA3.1(−) (Invitrogen) together with the gp120 signal peptide using T4 DNA ligase (Fermentas) as described above.

Site-directed mutagenesis was performed using the QuickChange multisite-directed mutagenesis kit (Stratagene, La Jolla, CA, USA) according to the protocol of the manufacturer. Eight amino acids in the isu-domain were substituted by Ala and Ala at positions 3 and 7 were substituted by Gly; two double mutations L1A + R4A and Q2A + L6A were also introduced to the cloned gp41. Using the vector expressing wt gp41 mutations were introduced to the glycosylation site N637T as described above.

To introduce the substitutions to the pNL4-3, the env fragment between nucleotides 7489 and 8526 was amplified by PCR using the primers: forward 5’-catgtggcaggaagtagg-3’ (position7489-7506) and reverse 5’-aagcggtggtagctgaag-3Â´ (position 8509–8526) and cloned in pTARGET (Promega, Madison, WI, USA). Site-directed mutagenesis was performed as described above. Plasmids with substitutions in the isu-domain were cleaved with Bam H1-Bse J1 and ligated using T4 DNA ligase (Promega) to pNL4-3 previously cleaved with Bam H1-Bse J1 (Fermentas). After ligation and transformation plasmids were isolated using a Wizard Plus SV Miniprep Kit (Promega) and Pure Yield Plasmid Midiprep System (Promega) and verified by restriction analysis and sequencing. In order to reduce the endotoxin level below 0.3 EI/ml, plasmids were purified twice using the MiraClean Endotoxin Removal kit (Mirus Bio, Madison, WI, USA). Low level of bacterial endotoxin in plasmids was confirmed by the LAL QCL-1000 test (Lonza, Verviers, Belgium).

### Transfection of cells, recovery of proteins from cell supernatants, and deglycosylation

293 T cells were washed three times with DMEM followed by culturing the cells in FCS-free medium containing as supplement 10% of Liforcell (Lifeblood media supplement, USA) to eliminate FCS proteins and allowed later concentration of gp41. 5-6 × 10^5^ cells were transfected with 3 μg of plasmid using 12 μl of TransIT-293 transfection reagent (Mirus Bio). 48 hrs post transfection the supernatants were harvested and centrifuged at 3000 g for 10 minutes and at 10000 g for 10 minutes. The supernatants were filtered through a 0.22 μm filter (Millipore GmbH, Germany) and concentrated 20–40 times using Vivaspin 4 concentrators (Sartorius Stedim Biotech GmbH, Göttingen, Germany) or U-tube concentrators (Novagen, EMD, Darmstadt Germany). The amounts of gp41 in cell supernatants were measured by Western blot analysis using as a reference serial dilution of T20 and the mab 2F5 for detection. To analyse glycosylation of the proteins, they were treated with PNGase F according to the protocol of the supplier (New England Biolabs, Inc., Ipswich, MA USA) and examined by SDS-PAGE/Western blot.

### Immunofluorescence

293 T cells were seeded on poly-L-lysine coated glass slides one day before transfection and transfected as described above. 48 hours post-transfection cells were washed with PBS and fixed for 10 minutes with 2.5% paraformaldehyde. Then the cells were washed twice with phosphate-buffered saline (PBS), permeabilised with 0.5% Triton X-100 (10 minutes), washed three times with PBS, blocked for 45 min with blocking buffer (1x PBS, 1% glycine, 0.1% Triton X-100) at room temperature and incubated with the mab 2F5 (20 μg/ml) in blocking buffer for 1 hour at room temperature. Cells were washed 3 times in PBS with 0.1% Triton X-100, stained with secondary anti-human Alexa Fluor 488 conjugate (Invitrogen), diluted 1:200 in blocking buffer and incubated for 1 hour at room temperature in the dark. The cells were washed 3 times with PBS and 0.1% Triton X-100, 3 times with PBS and analysed using an LSM 510 (Carl Zeiss, Oberkochen, Germany) confocal microscope using the LSM 5 Image Examiner software (Carl Zeiss).

### SDS-PAGE, native PAGE and Western blot analysis

Electrophoresis was performed in Tris-Glycine 4%–20% gradient gels using SDS Tris-Glycine sample buffer (Novex, Life technologies, Carlsbad, CA, USA). Native PAGE was performed in 4-20% Tris-Glycine gels using Tris-Glycine native running buffer (Novex) and samples in SDS free loading buffer were not heated before run. Proteins were transferred onto Protran BA83 0.2 μm membrane (Whatman GmbH, Dassel, Germany) at 45 V for 2 hours. Membranes were blocked with 6% skimmed milk in PBS with 0.1% Tween 20 (block buffer) for 3 hours at room temperature or overnight at 4°C, incubated for 2 hours at room temperature with serum from HIV-1 infected individuals or anti-gp41 mab 2F5 diluted in blocking buffer (1 μg/ml). After five times (5 min each) washing in PBS with 0.1% Tween 20 (PBS-Tween) the membranes were incubated for 1 hr with anti-human IgG-HRP conjugate (Dako Laboratories, Glostrup, Denmark) diluted 1:10000 in blocking buffer. The membranes were washed five times (5 minutes each) in PBS-Tween, treated for 1 minute with Pierce ECL Western blotting substrate (Pierce, Rockford, IL, USA) and exposed to CL-XPosure film (Thermo Scientific, Rockford, IL, USA). To detect antibodies against gp41 the recombinant ectodomain of gp41 produced in *E. coli* was used as antigen.

### Peptides and peptide polymers

Synthetic peptides corresponding to the immunosuppressive domain of gp41 of HIV-1 (aa 574–592 [GenBank: K03455]), (KQLQARILAVERYLKDQQL) (Figure 1) were produced by Genaxxon BioScience GmbH, (Ulm, Germany) or JPT, Jerini, (Berlin, Germany). Homopolymers of the peptides were produced by cross-linking with EDC and NHS (Pierce) as recommended by the supplier. T-20 (Fuzeon, aa 638–673, GeneBank: K03455, YTSLIHSLIEESQNQQEKNEQELLELDKWASLWNWF, Roche, Mannheim, Germany) was used as reference for the estimation of the gp41 amount in cell supernatants, virus particles and as antigen in ELISA.

### Human PBMCs

PBMCs were isolated from the blood of healthy donors by Ficoll-Hypaque (PAA Laboratories, Linz, Austria) density centrifugation using Leucosep tubes (Greiner Bio-one GmbH, Frickenhausen, Germany). Cells were washed and dissolved in complete RPMI 1640 medium containing 10% FCS, (Biochrome AG, Berlin, Germany) L-glutamine and antibiotics. A batch of FCS was selected which does induce only very low amount of IL-10. 1x10^5^ of PBMCs in 100 μl were mixed with 50 μl of gp41 protein (18 ng), peptide (12.5 μl) or virus (1.25 ng) and with 50 μl of complete RPMI medium. The cells were incubated at 37°C for 20-24 hours and cytokine release was measured by ELISA.

### ELISA for IL-6 and IL-10

Supernatants from PMBCs (1 × 10^5^ cells/well) either untreated or treated for 24 hours with the peptide polymers, gp41 or inactivated HIV-1 were collected, centrifuged at 2000 g for 10 min and tested by ELISA. The ELISAs were performed in triplicates according to the protocols of the suppliers of the IL-6 and IL-10 kits (BD Biosciences, San Diego, CA, USA). T20 ELISA was performed in 96 wells with 100 ng of the peptide per well.

### RNA isolation from PBMCs

RNA was isolated from donor PBMCs exposed to the supernatants containing wt gp41 or mutated gp41. In total, 1.8 × 10^6^ cells were used for RNA isolation using the RNeasy kit (Qiagen, Hilden, Germany). The RNA concentration was measured using a NanoDrop spectrometer ND-100 (PEQLAB Biotechnologie GmbH, Erlangen, Germany), and RNA specimens were used immediately in RT-PCR or kept at −80°C before use.

### One-step real-time quantitative PCR

One-step real-time RT-PCRs were established for human IL-6, IL-10, MMP-1 and FCN-1 ( Additional file 4), and duplex PCRs were performed using GAPDH for normalisation (ΔC_t_ = C_t_ gene of interest – C_t_ GAPDH). Total RNA was isolated as described above. Primers and probes for PCR were selected using the Sigma Genosys Probe Design Program and obtained from this company. 50 ng of RNA were used for amplification. Quantitative PCR was performed in triplicates in 25 μl (total volume) using 0.5 μl of polymerase from Super Script III Platinum one-step qRT-PCR kit (Invitrogen).

The thermal cycling profile for IL-10 was the following: 50°C/10 minutes and (95°C/15 s-56°C/30s-72°C/30s) 40 cycles, hold at 4°C. The thermal cycling profile for IL-6, FCN 1 and MMP-1 was the following: 50°C/10 minutes and (95°C/15 s-60°C/1 min) 45 cycles, hold at 4°C. Reporter fluorescence was measured using an Mx4000 Multiplex Quantitative PCR System (Stratagene, La Jolla, CA, USA) and evaluated using the 2^-ΔΔCT^ method [49].

### Virus preparations

Briefly, 293 T cells (1 × 10^6^) were transfected with 3 μg of each - pNL4-3, pNL4-3(A2) and pNL4-3(A2 + A6) using 4 μl/μg of Mirus TransIT-293 transfection reagent as described above. 48 hrs post transfection the supernatants were harvested, centrifuged at 3000 g for 10 min and at 10,000 g for 10 minutes. Supernatants were filtered through 0.45 μm filter (Millipore), centrifuged through a 20% sucrose cushion at 32000 rpm (rotor SW 50.1Ti, Beckman, Ireland) for 3 hours. Viruses were collected in PBS and used immediately or stored at -80°C until use. Virus preparations were added to PBMCs after six cycles of freeze-thawing procedure.

### Virus titration in TZM-bl cells

Infectivity was estimated in TZM-bl cells using 96-well plates and supernatants from two independent transfection experiments. 4 × 10^3^ cells per well were plated and incubated overnight at 37°C. On the next day when the cell monolayer was about 40-50% confluent, the medium was removed. 10 μl of virus-containing supernatants from transfected cells were diluted in 90 μl of complete medium and added to the TZM-bl cells. Titration was performed in triplicates by serial tenfold dilution. After 24 hours the medium was replaced; and 24 hours later, cells were washed twice with PBS and fixed with 2% paraformaldehyde for 5 minutes. Cells were washed twice with PBS and stained with X-gal (0.5 mg/ml) in PBS containing 5 mM K-ferric cyanide and 5 mM K-ferro cyanide and 2 mM MgCl_2_. Cells were incubated at room temperature in the dark, and on the next day blue-stained (β-Gal+) cells were counted using light microscope. Groups of blue-stained cells were counted as single foci of infection and wells containing >5 blue-stained cells were used for calculation.

### Immunisation studies

Wild-type gp41 and gp41 with the mutation Q2A in the isu domain both with and without the cytoplasmatic tail were produced by transfected 293 T cells and proteins were isolated from the supernatants of cells and concentrated as described above. Four rats were immunised subcutaneously with wt gp41 and mutated gp41 (200 ng per animal/per immunisation/boost), using complete (for immunisation) and incomplete (for boost immunisation) FreundÂ´s adjuvants. Sera were taken after the second boost and analysed in an ELISA (dilution 1:200) for antibodies binding to T20 and in a Western blot analysis with the ectodomain of gp41. Three rats were immunised with gp41ΔCT and three rats with mutated gp41ΔCT (Q2A) in the same way as described above. In these experiments 250 ng of antigen per animal/immunisation or boost were used. The animals were immunised according the German Animal Protection Law. The Landesamt für Gesundheit und Soziales Berlin reviewed and approved the protocol of the animal study (Approval number H0201/02).

### DNA analysis, protein structure analysis and protein calculation

The programs DNAStar Lasergene version 10, Clone Manager Professional version 10 and PsiPred server (http://http:/bioinf.cs.ucl.ac.uk/psipred/) were used for analysis of the nucleotide sequences and predictions of protein conformation. The calculation of gp41 in virus particles was based on our measurements performed by Western blot analysis with serial dilutions of reference peptide (T20). Estimation of the gp41 in association with virus particles was based on the known infectious titre and the estimated amount of gp41 using reference peptide (T20); and the infectious titre of the virus and also on published data [50].

### Statistical analysis

Medians for each triplicate donor PBMCs cytokine responses to gp41 (wt and mutants) were calculated, and their statistical significance was assessed using Student’s *t* test. P values of samples were calculated versus the wt gp41. Then, medians of the released IL-10 from each donor PBMCs were taken as basis data, and new medians encompassing all donors PBMCs responding to a particular protein were estimated. P values of these medians were calculated versus the wt median.

### Ethical statement

The use of human blood has been approved by the ethical commission at the Medical Faculty of the Humboldt University Berlin. Written informed consent was provided by study participants.

## Abbreviations

AIDS: Acquired immunodeficiency syndrome; FCN1: ficolin 1; HIV-1: Human immunodeficiency virus type 1; IL-6: Interleukin-6; IL-10: Interleukin-10; mab: Monoclonal antibody; MMP-1: Metalloproteinase 1; PBMCs: Peripheral mononuclear cells; wt: Wild-type.

## Competing interests

The authors declare that they have no competing interests.

## Authors’ contributions

VM planned, designed and together with AM performed the experiments on recombinant and mutated gp41 and replication-competent HIV-1 with mutated gp41. MS performed real time PCR experiments. JD organised and supervised the study.VM and JD wrote the manuscript. All authors read and approved the final manuscript.

## Supplementary Material

Additional file 1 **Detection of non-glycosylated and glycosylated forms of wt gp41 and wt gp41**Δ**CT.(A)** 293 T cells were transfected with the vector encoding wt gp41 (lane 1), and the backbone vector pcDNA3.1(−) as a negative control (lane 2). Cells were lysed 48 hours later, and proteins were examined by Western blot analysis. Non-glycosylated (p35) and glycosylated (gp39-gp41) forms are marked by arrow heads. **(B)** Comparative SDS-PAGE/Western blot analysis of supernatants from 293 cells transfected with the backbone vector (lane 1), the vector expressing the wt gp41 (lane 2) and gp41 harboring a mutation in one glycosylation site (N637T) (lane 3). **(C)** Comparative SDS-PAGE/Western blot analysis of lysates from 293 T cells grown in FCS-free medium expressing wt gp41ΔCT (lane 1), mutated gp41ΔCT(2A) (lane 2), transfected with the backbone vector pcDNA3.1(−) (lane 3). In parallel supernatants from non-transfected cells (lane 4), from cells transfected with the backbone vector pcDNA3.1(−) (lane 5), from cells producing wt gp41ΔCT (lane 6) and mutated gp41ΔCT(2A) (lane 7) were analysed. All supernatants were concentrated 20 times, and two glycosylated forms (about gp33 and gp36) are marked by arrow heads. Click here for file

Additional file 2 **Induction of IL-10 release by wt gp41 and wt gp41**Δ**CT and mutations in the isu domain abrogating IL-10 release.** Cytokine release was studied in PBMCs of donor 2 after exposure to wt gp41ΔCT, wt gp41 and gp41 with mutations 1A, 2A. The mock control comes from the supernatant of cells transfected with the empty vector. P values were estimated in comparison to wt gp41. Click here for file

Additional file 3 **Statistical significance of the IL-10 release from PBMCs of each of the four donors (A) and of the IL-6 release from PBMCs of donor 4 (B) as shown in Figure**3**.** The P values were estimated in comparison to the wt gp41. Click here for file

Additional file 4 **Statistical significance of the IL-10 release (A) and of the transcriptional activation of IL-6 (B), MMP-1 (C) and FCN1 (D) as shown in Figure**4**.** P values were estimated in comparison to wt gp41. Click here for file

Additional file 5 List of the primers and probes used for real-time PCR. Click here for file
