# Supplementary figures and images for: Single mutations in the transmembrane envelope protein abrogate the immunosuppressive property of HIV-1
Source: Retrovirology. 2012 Aug 13;9:67. doi: 10.1186/1742-4690-9-67 (PMC3464125; doi:10.1186/1742-4690-9-67)

## Slide 1
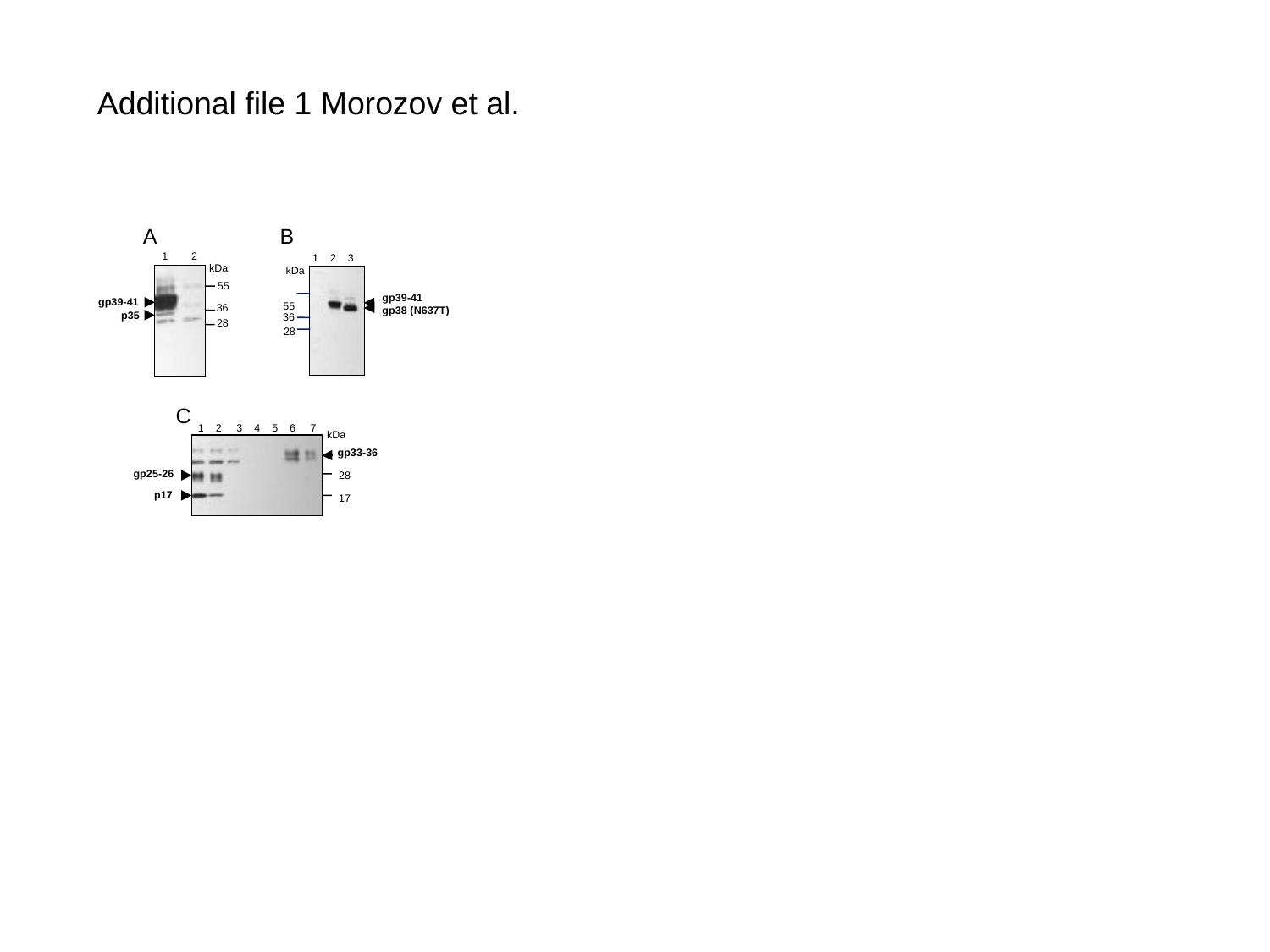

Additional file 1 Morozov et al.
 A B
 1 2
1 2 3
kDa
kDa
 55
55
gp39-41
gp38 (N637T)
 gp39-41
 36
36
 p35
 28
28
C
 1 2 3 4 5 6 7
gp25-26
28
p17
17
gp33-36
kDa

Supplement: Additional file 1 — Detection of non-glycosylated and glycosylated forms of wt gp41 and wt gp41ΔCT.(A) 293 T cells were transfected with the vector encoding wt gp41 (lane 1), and the backbone vector pcDNA3.1(−) as a negative control (lane 2). Cells were lysed 48 hours later, and proteins were examined by Western blot analysis. Non-glycosylated (p35) and glycosylated (gp39-gp41) forms are marked by arrow heads. (B) Comparative SDS-PAGE/Western blot analysis of supernatants from 293 cells transfected with the backbone vector (lane 1), the vector expressing the wt gp41 (lane 2) and gp41 harboring a mutation in one glycosylation site (N637T) (lane 3). (C) Comparative SDS-PAGE/Western blot analysis of lysates from 293 T cells grown in FCS-free medium expressing wt gp41ΔCT (lane 1), mutated gp41ΔCT(2A) (lane 2), transfected with the backbone vector pcDNA3.1(−) (lane 3). In parallel supernatants from non-transfected cells (lane 4), from cells transfected with the backbone vector pcDNA3.1(−) (lane 5), from cells producing wt gp41ΔCT (lane 6) and mutated gp41ΔCT(2A) (lane 7) were analysed. All supernatants were concentrated 20 times, and two glycosylated forms (about gp33 and gp36) are marked by arrow heads. [file 1742-4690-9-67-S1.ppt]

## Slide 1
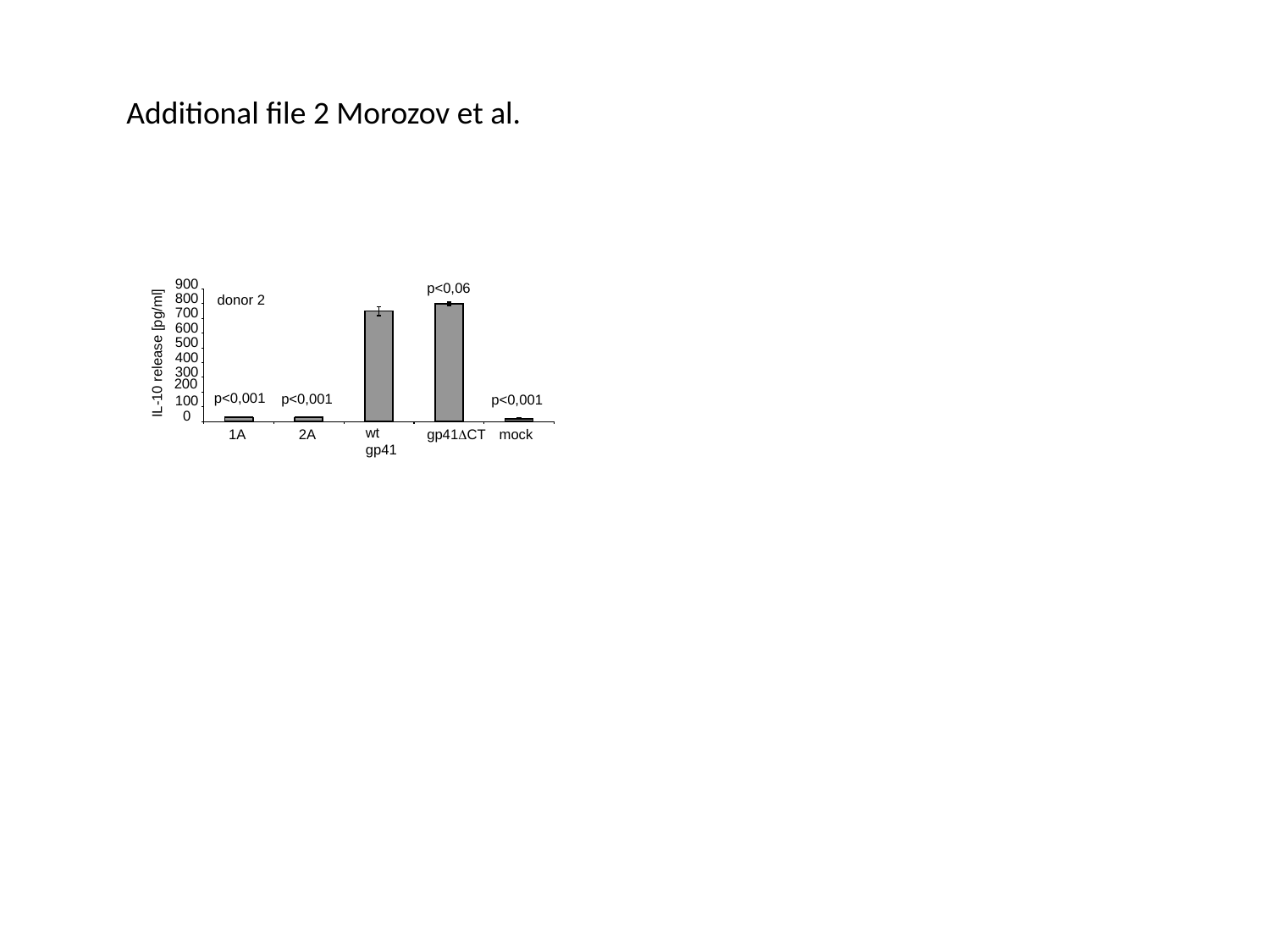

Additional file 2 Morozov et al.
p<0,06
900
800
donor 2
700
600
IL-10 release [pg/ml]
500
400
300
200
p<0,001
p<0,001
p<0,001
100
0
wt
gp41
 1A
 2A
gp41DCT
mock

Supplement: Additional file 2 — Induction of IL-10 release by wt gp41 and wt gp41ΔCT and mutations in the isu domain abrogating IL-10 release. Cytokine release was studied in PBMCs of donor 2 after exposure to wt gp41ΔCT, wt gp41 and gp41 with mutations 1A, 2A. The mock control comes from the supernatant of cells transfected with the empty vector. P values were estimated in comparison to wt gp41. [file 1742-4690-9-67-S2.pptx]

## Slide 1
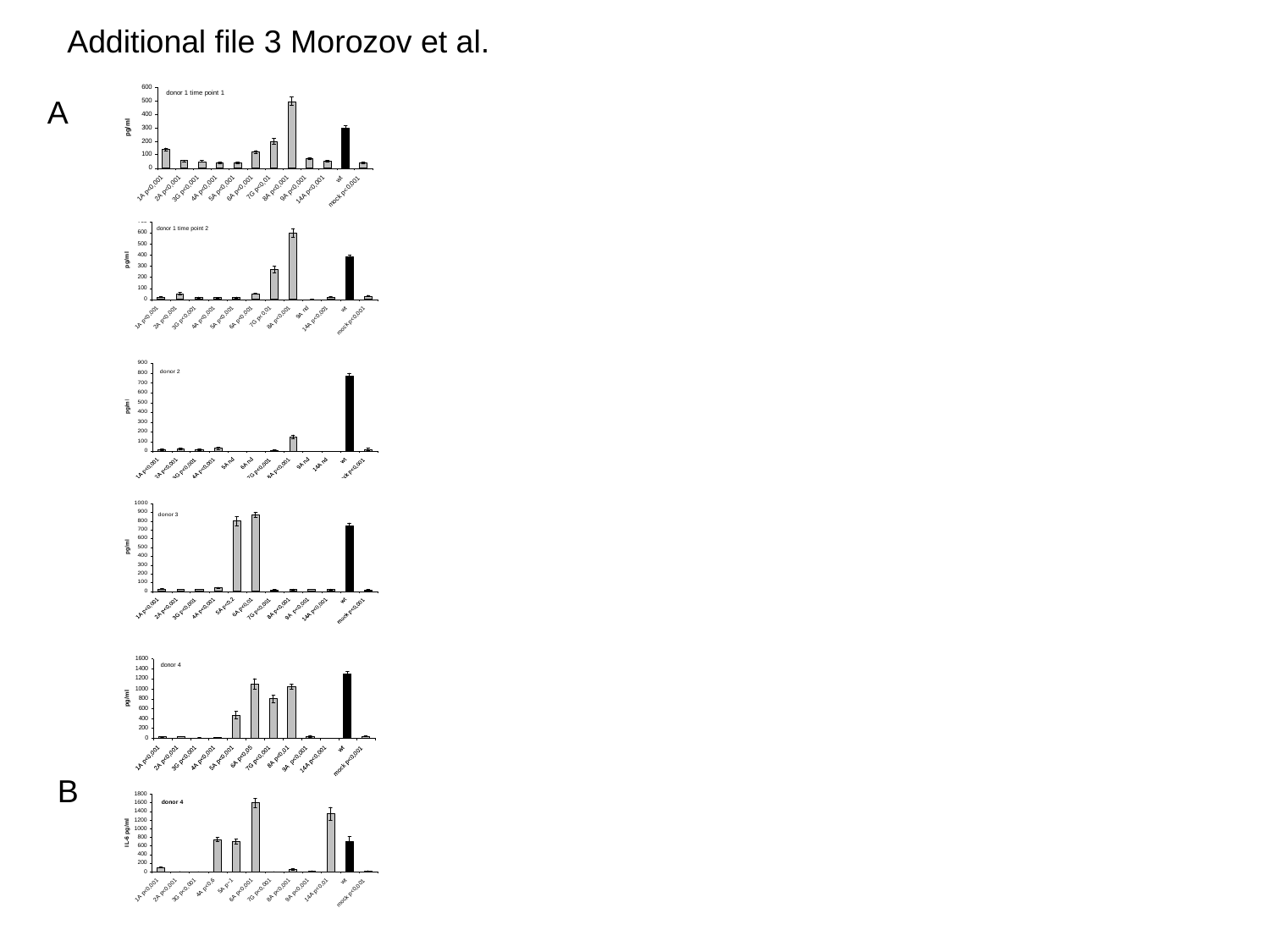

Additional file 3 Morozov et al.
A
B

Supplement: Additional file 3 — Statistical significance of the IL-10 release from PBMCs of each of the four donors (A) and of the IL-6 release from PBMCs of donor 4 (B) as shown in Figure3. The P values were estimated in comparison to the wt gp41. [file 1742-4690-9-67-S3.ppt]
