# Supplementary material for: Single mutations in the transmembrane envelope protein abrogate the immunosuppressive property of HIV-1
Source: Retrovirology. 2012 Aug 13;9:67. doi: 10.1186/1742-4690-9-67 (PMC3464125; doi:10.1186/1742-4690-9-67)
Supplement: Additional file 4 — Statistical significance of the IL-10 release (A) and of the transcriptional activation of IL-6 (B), MMP-1 (C) and FCN1 (D) as shown in Figure4. P values were estimated in comparison to wt gp41. [file 1742-4690-9-67-S4.pptx]

## Slide 1
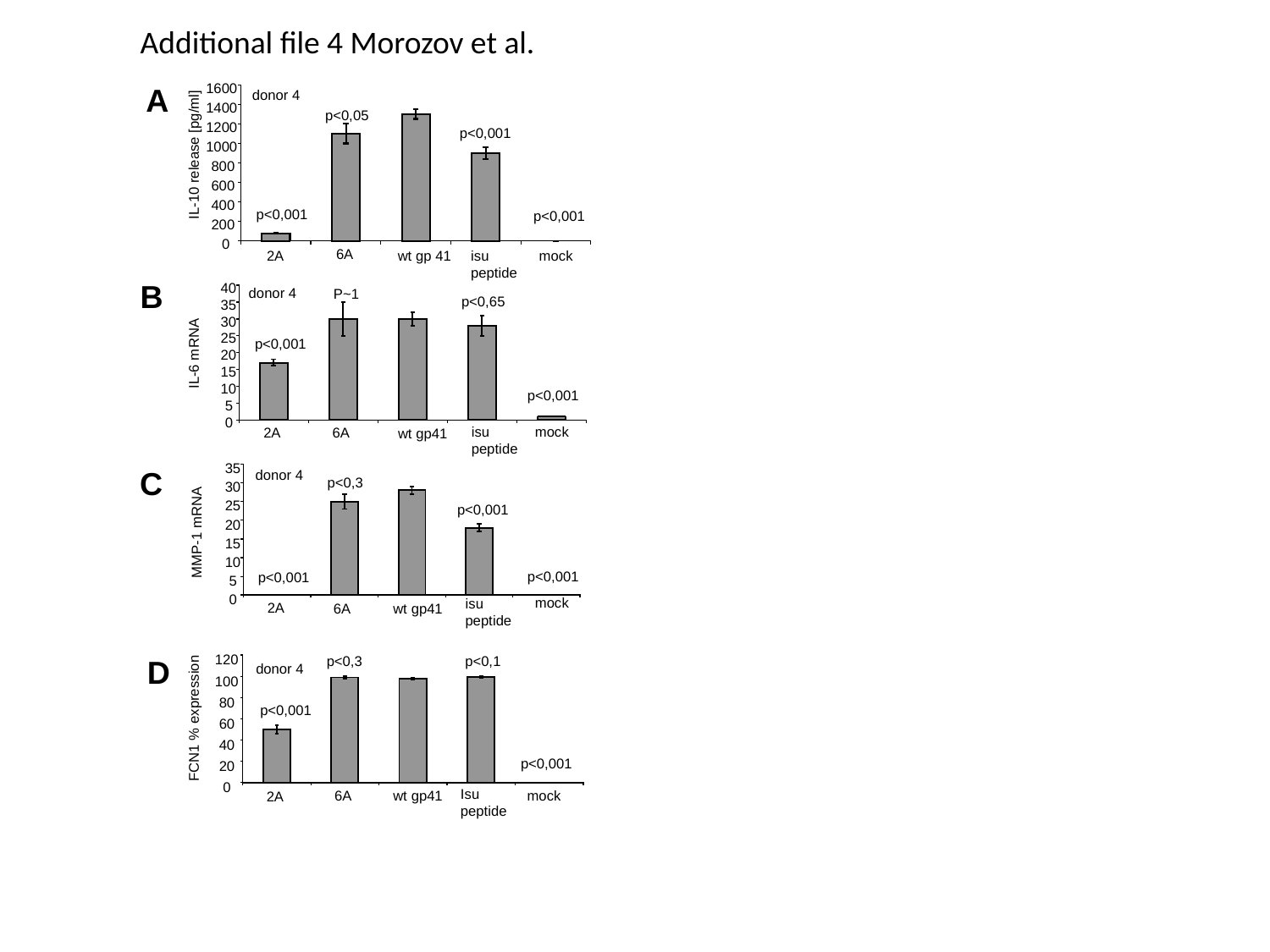

Additional file 4 Morozov et al.
A
B
C
D
1600
donor 4
1400
1200
1000
800
600
400
200
0
6A
2A
wt gp 41
isu
peptide
mock
40
donor 4
35
30
25
IL-6 mRNA
20
15
10
5
0
isu
peptide
mock
6A
2A
wt gp41
35
donor 4
30
25
20
MMP-1 mRNA
15
10
5
0
mock
isu
peptide
2A
6A
wt gp41
FCN1 % expression
120
donor 4
100
80
60
40
20
0
Isu
peptide
6A
wt gp41
mock
2A
p<0,05
IL-10 release [pg/ml]
p<0,001
p<0,001
p<0,001
P~1
p<0,65
p<0,001
p<0,001
p<0,3
p<0,001
p<0,001
p<0,001
p<0,3
p<0,1
p<0,001
p<0,001
