# Supplementary material for: Single mutations in the transmembrane envelope protein abrogate the immunosuppressive property of HIV-1
Source: Retrovirology. 2012 Aug 13;9:67. doi: 10.1186/1742-4690-9-67 (PMC3464125; doi:10.1186/1742-4690-9-67)
Supplement: Additional file 5 — List of the primers and probes used for real-time PCR. [file 1742-4690-9-67-S5.doc]

**Additional file 5 Primer and probes for the real-time RT-PCR**

| **Probe** | | | |
| --- | --- | --- | --- |
| Gene | Accession nr | Sequence | nt - nt |
| hsGAPDH | NM_002046.3 | Hex-CTTCACCACCATGGAGAAGGCTGGG  [3BQ1] | 405-429 |
| hsIL-10 | NM_000572.2 | 6Fam-TCTTGTCTCTGGGCTT[3BQ1] | 1015-1030 |
| hsIL-6 | NM_000600.3 | 6Fam-TGTTACTCTTGTTACATGTCTCCTTTCTCAGGGCT[3BQ1] | 311-345 |

| hsMMP-1 | NM_002421.3 | 6Fam-CTGGGCTGTTCAGGGACAGAA[3BQ1] | 1187-1207 |
| --- | --- | --- | --- |

| hsFCN1 | NM_002003.3 | 6Fam-TATTTCCTGAGCGGCTGGCACA[BHQ1] | 465-486 |
| --- | --- | --- | --- |
| Primer | | | |
| Gene | Accession nr | Sequence | nt – nt |
| hsGAPDH | NM_002046.3 | 5´GGCGATGCTGGCGCTGAGTA3´ | 364-386 |
|  |  | 3´ AGCAGTACCCACACTTGGT 5´ | 494-512 |
| hsIL-10 | NM_000572.2 | 5´CCACGCTTTCTAGCTGTT3´ | 966-983 |
|  |  | 3´ AATCCTTCTCTTTGGTCCCTC 5´ | 1058-1078 |
| hsIL-6 | NM_000600.3 | 5´GGTACATCCTCGACGGCATCT3´ | 289-309 |
|  |  | 3´ CACTTTCGTCGTTTCTCCGTG 5´ | 349-369 |

| hsMMP-1 | NM_002421.3 | 5´CATCCAAGCCATATATGGACG3´ | 908-928 |
| --- | --- | --- | --- |
|  |  | 3´TCTCTTAAAACTGAGAGGTCT5´ | 1498-1518 |
| hsFCN1 | NM_002003.3 | 5’CTGCAAGGACCTGCTAGACC3’ | 440-459 |
|  |  | 5’ CTACCTACCGAGACACCTGA3’ | 572-591 |
